# Single-Cell RNA Sequencing Reveals Cellular and Transcriptional Changes Associated With Traumatic Brain Injury

**DOI:** 10.3389/fgene.2022.861428

**Published:** 2022-06-30

**Authors:** Jin Xing, Li Ren, Hao Xu, Liang Zhao, Zhi-Han Wang, Guang-Dong Hu, Zi-Long Wei

**Affiliations:** Department of Neurosurgery, Shanghai Pudong Hospital, Fudan University Pudong Medical Center, Shanghai, China

**Keywords:** traumatic brain injury, single-cell RNA sequencing, inflammation, cilium movement, excitotoxicity

## Abstract

Traumatic brain injury (TBI) is currently a substantial public health problem and one of the leading causes of morbidity and mortality worldwide. However, the cellular and transcriptional changes in TBI at single-cell level have not been well characterized. In this study, we reanalyzed a single-cell RNA sequencing (scRNA-seq) dataset of mouse hippocampus to identify the key cellular and transcriptional changes associated with TBI. Specifically, we found that oligodendrocytes were the most abundant cell type in mouse hippocampus, and detected an expanded astrocyte population, which was significantly activated in TBI. The enhanced activity of inflammatory response-related pathways in the astrocytes of TBI samples suggested that the astrocytes, along with microglia, which were the major brain-resident immune cells, were responsible for inflammation in the acute phase of TBI. Hormone secretion, transport, and exocytosis were found upregulated in the excitatory neurons of TBI, which gave us a hint that excitatory neurons might excessively transport and excrete glutamate in response to TBI. Moreover, the ependymal subpopulation C0 was TBI-specific and characterized by downregulated cilium movement, indicating that the attenuated activity of cilium movement following TBI might decrease cerebrospinal fluid flow. Furthermore, we observed that downregulated genes in response to candesartan treatment were preferentially expressed in excitatory neurons and were related to pathways like neuronal systems and neuroactive ligand-receptor interaction, indicating that candesartan might promote recovery of neurons after traumatic brain injury *via* mediating neuroactive ligand-receptor interactions and reducing excitotoxicity. In conclusion, our study identified key cell types in TBI, which improved our understanding of the cellular and transcriptional changes after TBI and offered an insight into the molecular mechanisms that could serve as therapeutic targets.

## Introduction

Traumatic brain injury (TBI), which is caused by an external physical force, remains a leading cause of death and acquired disability worldwide (Pearn et al., 2017; Khellaf et al., 2019). Cognitive deficits can be observed during the post-injury phases of TBI, namely the acute phase, the subacute phase, and the chronic phase (Mayer et al., 2009; Pavlovic et al., 2019). TBI could inflict neuronal injury, and the underlying mechanisms has been widely pursued (Jamjoom et al., 2021).

Currently, more and more studies focus on the changes of cell types in response to TBI. Neuroinflammation is recognized as one of the hallmarks in TBI, which involves the activation of microglia or astrocytes and release of inflammatory factors within the brain (Lozano et al., 2015). A recent study has stated that microglia-mediated inflammation could result in adverse neuronal alterations at subacute and chronic time points after diffuse TBI, thus preventing TBI-induced cognitive changes, but many transcriptional responses during the acute phase seemed to be microglia-independent (Witcher et al., 2021), while another study demonstrated that inducing microglia turnover during the acute phase of TBI exerted neuroprotective effects, which was mediated *via* IL-6 trans-signaling (Willis et al., 2020). Among brain resident glial cells, astrocyte is the most abundant within brain (Yang and Wang, 2015) and its activation involves excessive proliferation and release of inflammatory mediators and growth factors (Gorina et al., 2011; Zamanian et al., 2012; Paintlia et al., 2013). In addition, TBI could also result in oligodendrocyte death and increase the number of oligodendrocyte progenitor cell (Flygt et al., 2016) and cellular excitotoxicity of astrocytes within the brains (Sofroniew and Vinters, 2010), which are considered as key mediators in the pathophysiology of TBI. However, due to the complexity of TBI, the transcriptional changes of cell types within brain in response to TBI have not been well characterized.

Utilizing single-cell RNA sequencing (scRNA-seq) technology, TBI-related cellular and molecular adaptations have been captured and investigated at single-cell resolution. In mTBI mice, changes during the acute phase were reported in the cell population and the global transcriptome patterns of a variety of cell types, such as ependymal cells, microglia, endothelial cells, neurons, and oligodendrocytes (Arneson et al., 2018). In this study, we conducted an in-depth analysis of the scRNA-seq data from the previous study (Arneson et al., 2018), and anticipated to recognize the key cellular and transcriptional changes associated with TBI.

## Materials and Methods

### Single-Cell RNA-Seq Data Collection and Preprocessing

The single-cell RNA sequencing (scRNA-seq) data of three Sham and three TBI samples were collected from Gene Expression Omnibus (GEO) under accession number GSE101901 (Arneson et al., 2018). The count matrix of six samples were used for the downstream analysis using R Seurat package (Stuart et al., 2019). Cells that expressed less than 200 genes were eliminated and genes detected in less than three cells were filtered.

### Multiple Dataset Integration and Dimensionality Reduction

The multiple datasets were integrated by R Harmony (Korsunsky et al., 2019) package. Maximum number of rounds to run Harmony was set to 20. (Korsunsky et al., 2019). The remaining parameters were set to default values. The FindVariableFeatures in R Seurat was used to select the top 3,000 highly variable features. The counts for each cell are divided by the total counts for that cell and multiplied by the scale factor (10000), and then natural-log transformed. The expression levels displayed in all figures were natural-log-transformed. The cells were clustered at a resolution of 0.5. The Uniform Manifold Approximation and Projection (UMAP) method was applied to reduce the dimensionality and to visualize the cell clusters.

### Differentially Expressed Genes and Cell Type Annotation

The differentially expressed genes were identified by FindAllMarkers in R Seurat package (Stuart et al., 2019). The Wilcoxon rank sum test was used to measure the statistical significance of the difference between the cell types. For the convenience of calculation, the genes with an adjusted *p*-value < 0.05 and log2 fold change > 0.5 were considered as the DEGs. The cell types were annotated based on their marker genes from the earlier study (Arneson et al., 2018).

### Gene Set Overrepresentation Enrichment Analysis

The overrepresentation of terms from KEGG (https://www.genome.jp/kegg/), Reactome (Jassal et al., 2020) (https://reactome.org), WikiPathways (https://www.wikipathways.org/index.php/WikiPathways), and Gene Ontology (GO-bp, http://geneontology.org) in a specific list of genes was then investigated. The gene sets were obtained using R msigdbr package (https://cran.r-project.org/web/packages/msigdbr/index.html). The hypergeometric test was used to measure the degree of enrichment. The ORA was implemented in R clusterProfiler (Yu et al., 2012).

### Pseudo-Bulk RNA-Seq Data Analysis

The pseudo-bulk RNA-seq was used to identify the genes differentially expressed in a specified cell type between TBI and Sham samples following the previous study (Squair et al., 2021). The read count was aggregated per sample. Specifically, the sum of counts for each sample within each cell type was calculated to represent its gene expression level. Subsequently, R DESeq2 (Love et al., 2014) package was employed to identify the genes differentially expressed between TBI and Sham samples.

### Statistical Analyses

The statistical analyses were performed in R v4.1.0. Wilcoxon rank sum test was used in the two-sample or pairwise comparisons. The differences between the proportions were tested by Pearson’s chi-squared test, implemented in R prop. test. The asterisks of *, **, and *** represented the statistical significance at 0.05, 0.01, and 0.001, respectively.

## Results

### Identification of the Cell Types in Mouse Hippocampus at Single-Cell Level

**FIGURE 1:**
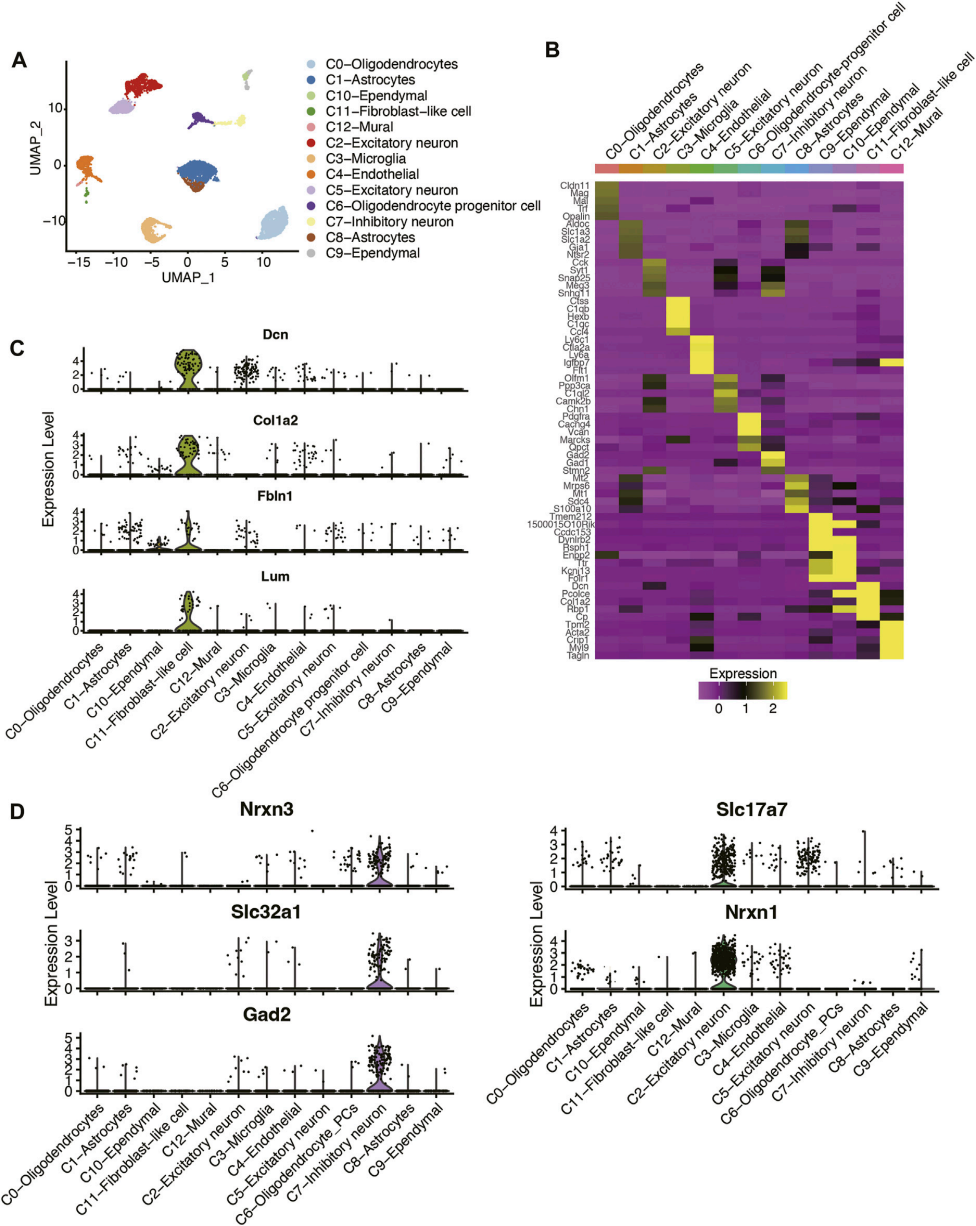
Classification and molecular characterization of the cell types in mouse hippocampus of TBI and Sham samples. **(A)** The UMAP analysis for the mouse hippocampus samples. Each point represents one cell, and the point colors represent the cell clusters. **(B)** The expression patterns of the cell type maker genes across the cell types. The expression levels of **(C)** fibroblast-like cell, **(D)** excitatory neuron, and inhibitory neuron marker genes across the cell types.

To identify the cell types in mouse hippocampus, we collected and re-analyzed the scRNA-seq data of three TBI and three Sham samples from the previous study (Arneson et al., 2018). The cells with low quality were removed if the percentage of reads mapping to mitochondrial genes was above 10% or the number of detected RNA features was less than 200. The six hippocampus samples were subsequently merged using Harmony algorithm, and the clusters were identified using a shared nearest neighbor (SNN) modularity optimization-based clustering algorithm (See Materials and methods). As shown in Figure 1A, the cells from the six samples were clustered into 13 clusters. Based on the expressions of cell markers reported by the earlier study (Arneson et al., 2018), we successfully annotated those cell types (Figure 1B), including astrocytes, endothelial cell, ependymal cell, excitatory neuron, fibroblast-like cell, inhibitory neuron, microglia, mural, oligodendrocyte-progenitor cells (OPCs), or oligodendrocyte. Notably, we also found that the fibroblast markers, such as *Dcn*, *Col1a2*, *Fbln1*, and *Lum*, were highly expressed in C11 cluster, which was termed as fibroblast-like cell (Figure 1C). Furthermore, two neuron clusters were characterized as excitatory and inhibitory neurons by the markers reported by previous studies (Bhattacherjee et al., 2019; Delile et al., 2019) (Figure 1D), respectively. These results indicated that the cell types in mouse hippocampus tissues could be well-characterized using the scRNA-seq data.

### Functional Characterization of Oligodendrocyte Subpopulations

**FIGURE 2:**
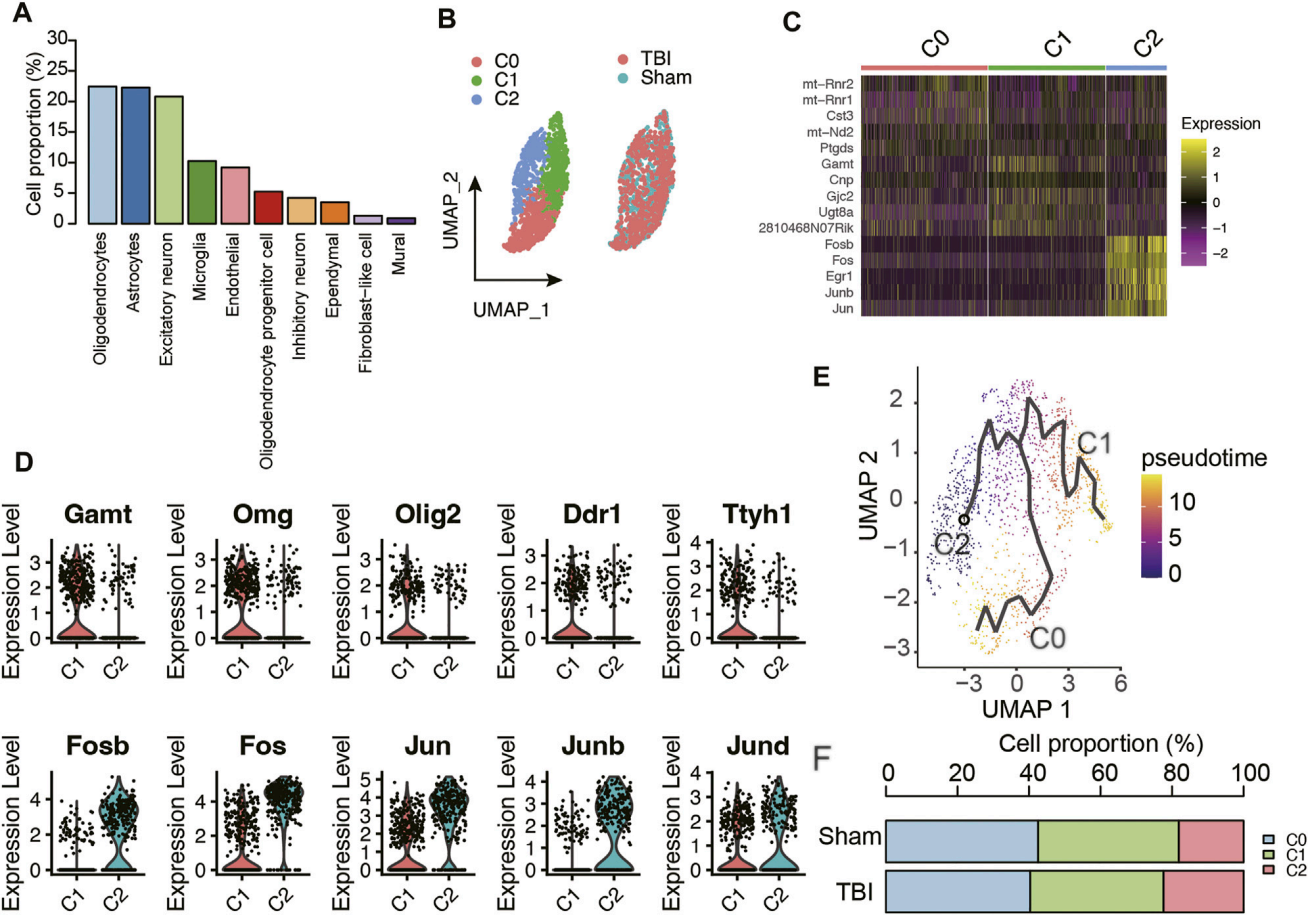
The molecular characteristics of oligodendrocyte subpopulations. **(A)** The proportion of cell types in the mouse hippocampus samples. **(B)** The sub-clustering analysis of oligodendrocytes. **(C)** The top-5 differentially expressed genes of the three oligodendrocyte subpopulations. **(D)** The marker genes of oligodendrocyte C1 and C2 subpopulations. **(E)** The trajectory analysis of the oligodendrocyte subpopulations. **(F)** The proportion of oligodendrocyte populations in TBI and Sham samples.

As the cell clusters were characterized by their marker genes, the adjacent clusters characterized as the same cell type were merged, and a total of 10 cell types in mouse hippocampus were identified (Figure 1A). Notably, oligodendrocyte, astrocyte, and excitatory neuron accounted for over 20% of the cells, followed by microglia, endothelial cell, oligodendrocyte progenitor cells, inhibitory neuron, ependymal cell, fibroblast-like cell, and mural (Figure 2A). Considering that oligodendrocytes were the most abundant cell type, we conducted sub-clustering analysis on this cell type and identified three sub-clusters within oligodendrocytes (Figure 2B). The differential gene expression analysis was conducted to identify the differentially expressed genes across the oligodendrocyte subclusters (Figure 2C), and the results suggested that the oligodendrocyte subclusters had significantly different transcriptional programs. Specifically, higher expression levels of mitochondrial genes like *mt-Rnr2*, *mt-Rnr1*, and *mt-Nd2* were observed in oligodendrocyte-C0 (Figure 2C), suggesting that this subcluster might be in an apoptotic state (Ilicic et al., 2016). Furthermore, the gene set enrichment analysis revealed that oligodendrocyte-C1 might be differentiated oligodendrocyte, as markers of mature oligodendrocyte such as *Gamt*, *Omg*, *Olig2*, *Ddr1*, and *Ttyh1* were exclusively expressed in this cluster (Figure 2D). The oligodendrocyte-C2 was observed to have higher expression levels of transcription factors such as *Fosb*, *Fos*, *Jun*, *Junb*, *Jund* (Figure 2D), which showed high similarity with the oligodendrocyte in intermediate early state as reported by the previous study (Marques et al., 2018). The trajectory analysis also indicated that the oligodendrocytes might develop from C2 to C1, eventually to C0 (Figure 2E). Though the proportions of these oligodendrocyte subclusters were not significantly altered in TBI (*t* test, *p*-value > 0.05), we still observed a relative increase in the proportions of C2 (TBI vs. Sham, 22.38% vs. 18.11%) and decrease in the proportions of C0 (TBI vs. Sham, 40.32% vs. 42.55%) and of C1 (TBI vs. Sham, 37.30% vs. 39.33%) in TBI, after merging the TBI or Sham replicates (Figure 2F). These results indicated that TBI might lead to an increase in the proportions of immature oligodendrocytes and a decrease in that of differentiated oligodendrocytes.

### Astrocytes Are Significantly Activated in Traumatic Brain Injury Tissues With an Expanded Population

**FIGURE 3:**
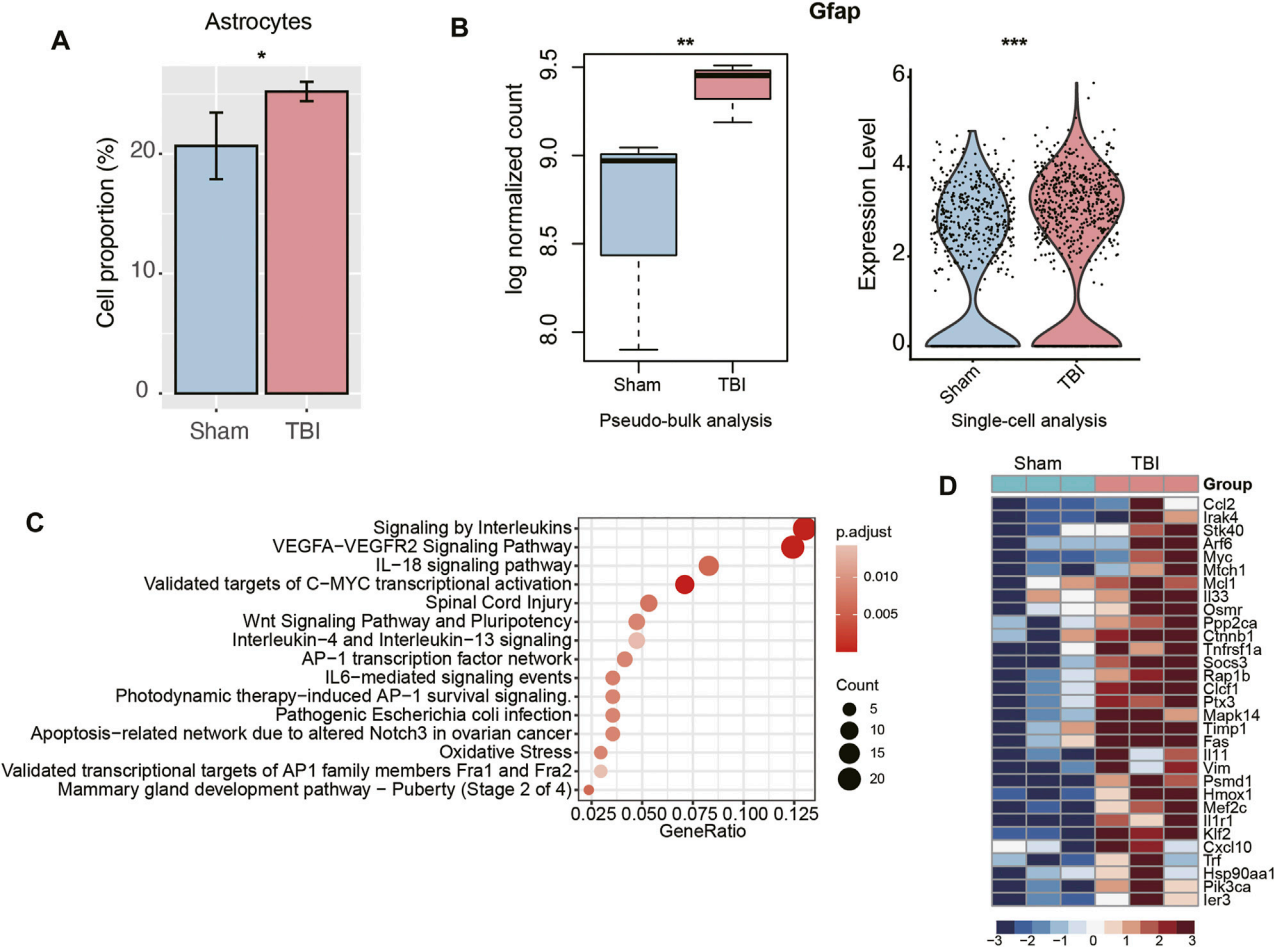
The transcriptional changes of astrocytes of TBI. **(A)** The relative proportions of astrocytes between TBI and Sham samples. **(B)** The differential expression levels of Gfap between TBI and Sham samples. The left and right panels illustrated the pseudo-bulk and single-cell analyses, respectively. **(C)** The pathways enriched by the upregulated genes in the astrocytes of TBI. **(D)** The key genes upregulated in the astrocytes of TBI.

To further identify the cell types with significantly altered populations, we compared the cell proportions of TBI group with those of Sham group. Remarkably, an expanded population of astrocytes was observed in TBI samples (Figure 3A, *p*-value < 0.05). To test whether astrocytes were activated in TBI, we examined the expression levels of glial fibrillary acidic protein (*Gfap*) in this cell type, as it was a marker of astrocyte activation and a hallmark of multiple central nervous system (CNS) pathologies (Sofroniew, 2009). Consistently, *Gfap* was upregulated in TBI, which was detected through both pseudo-bulk RNA-seq analysis and scRNA analysis (adjusted *p*-value < 0.05 and fold change > 1.5, Figure 3B). Moreover, we also investigated the biological processes enriched by the upregulated genes in TBI and found that inflammatory response-related pathways, including signaling by interleukins, IL18 signaling pathway, and IL4 and IL13 signaling pathways, were significantly enriched by those genes (Figure 3C). Notably, the cytokine *Ccl2*, chemokine *Cxcl10*, interleukins or interleukin receptors including *Il33*, *Il11*, and *Il1r* were upregulated in TBI samples (Figure 3D). These results indicated that astrocytes were significantly activated and associated with neuroinflammation in TBI.

### Excitatory Neurons Excessively Transport and Excrete Glutamate in Response to Traumatic Brain Injury

**FIGURE 4:**
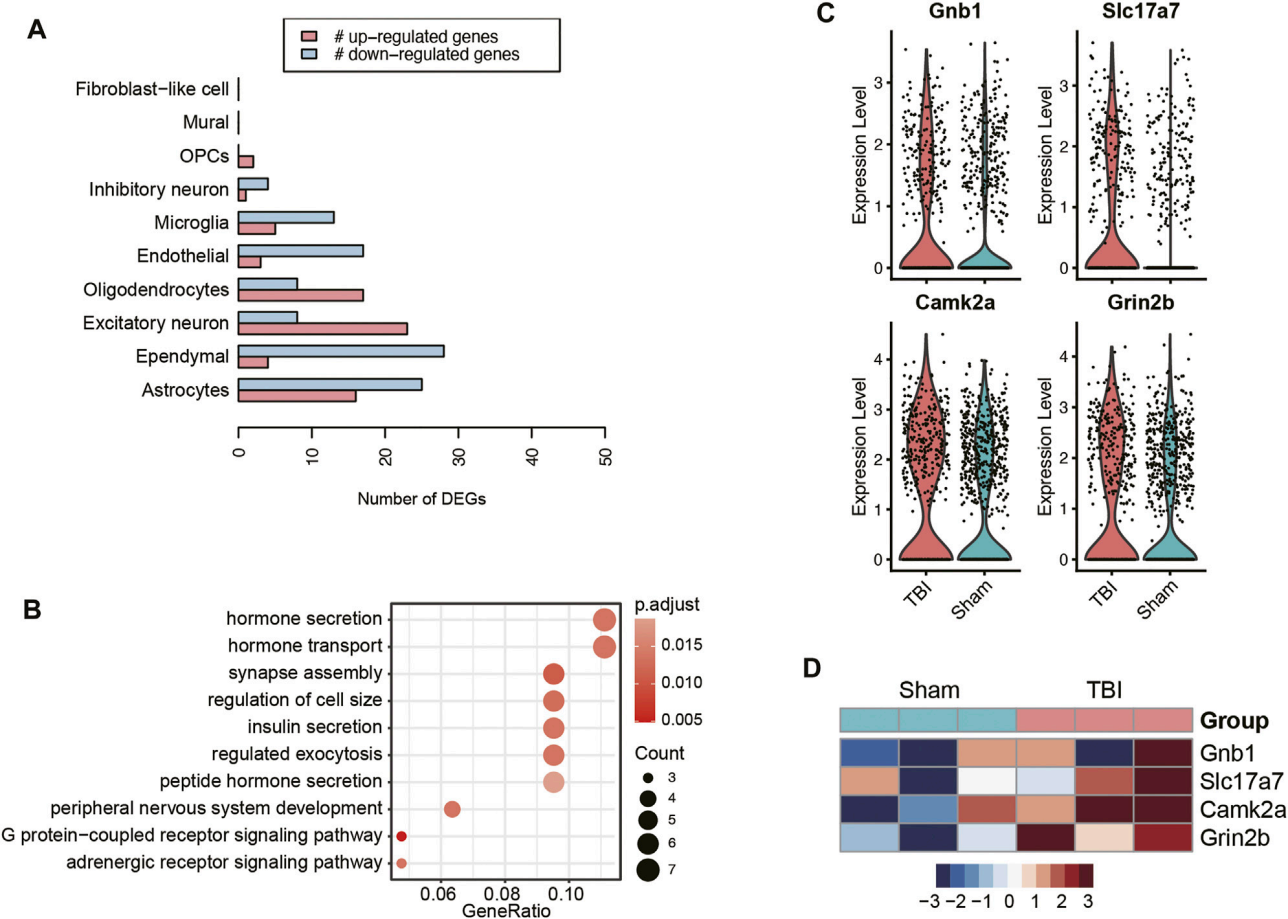
The transcriptional changes of excitatory neurons of TBI. **(A)** The number of upregulated and downregulated genes in each cell type of TBI. **(B)** The pathways enriched by the upregulated genes in the excitatory neurons of TBI. The key genes upregulated in the excitatory neurons of TBI, visualized at single-cell level **(C)** and pseudo-bulk level **(D)**.

As cellular excitotoxicity is a key mediator in the pathophysiology of TBI, we then investigated the transcriptional changes of excitatory neurons in TBI. The pseudo-bulk RNA-seq analysis revealed that excitatory neurons presented the highest number of upregulated genes (Figure 4A), indicating that the transcriptional upregulation in excitatory neuron was more predominant than other cell types. The gene set overrepresentation enrichment analysis (ORA) revealed that the upregulated genes in TBI were involved in hormone secretion, transport and exocytosis (Figure 4B). Differential expression analyses between excitatory neurons of TBI and Sham, which were based on the integration of single-cell and pseudo-bulk RNA-seq data, identified *Gnb1*, *Slc17a7*, *Camk2a*, and *Grin2b* as the key genes involved in glutamate transport and secretion (Figures 4C,D). These results further demonstrated that excitatory neurons excessively transported and excreted glutamate in response to TBI.

### Traumatic Brain Injury Affects Transition Metal Ion Homeostasis and Cilium Movement of Ependymal Cell

**FIGURE 5:**
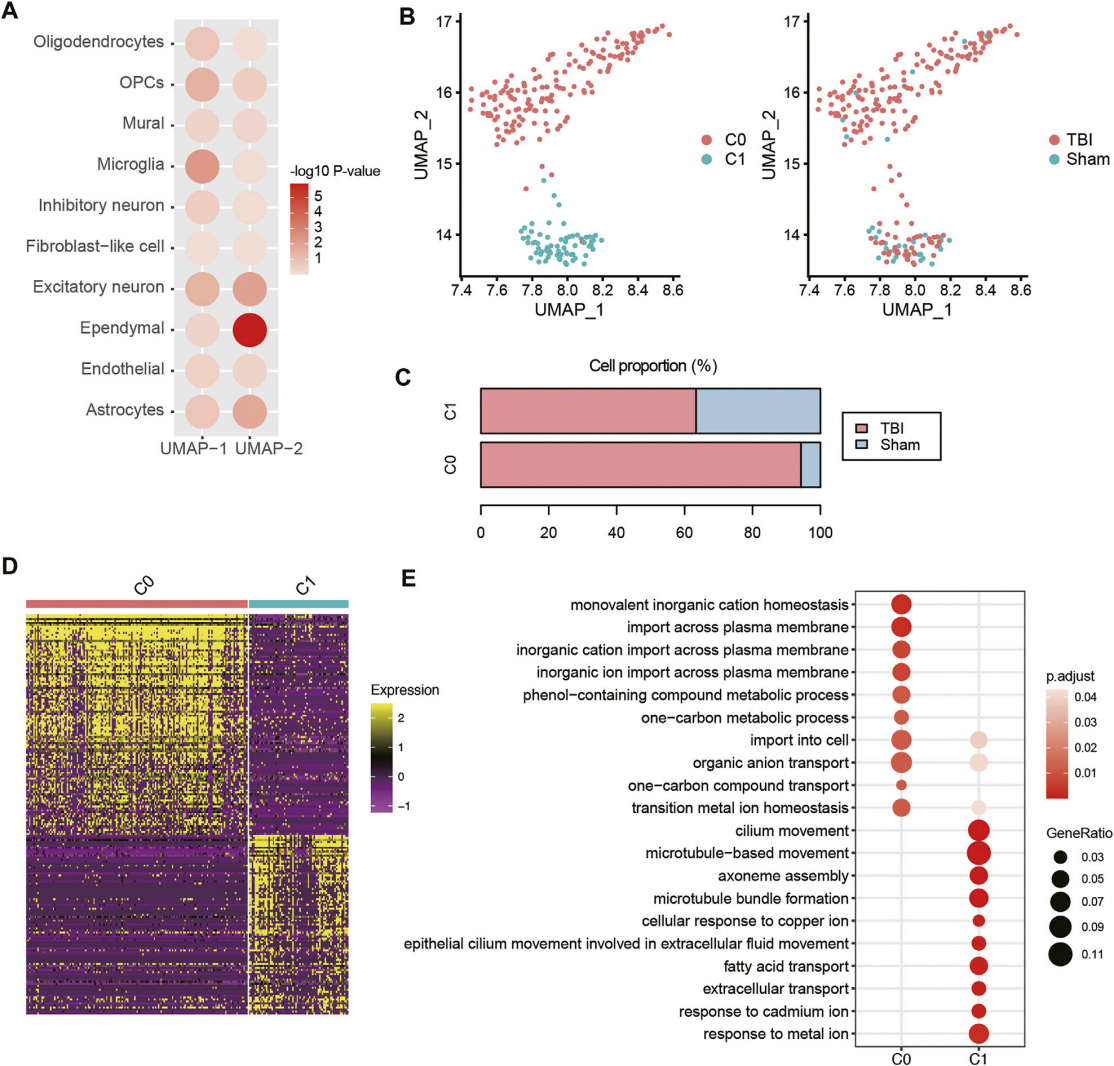
The transcriptional changes of ependymal cells of TBI. **(A)** The differential distribution of the top-two UMAP components (UMAP-1 and UMAP-2) between TBI and Sham. The Wilcoxon rank sum test was applied. **(B)** The sub-clustering of ependymal cells. **(C)** The proportion of ependymal cell populations in TBI and Sham samples. **(D)** The differential expression patterns of marker genes between the two ependymal cell subpopulations. **(E)** The pathways enriched by the marker genes of the two ependymal cell subpopulations.

To identify the TBI-related cell types that could be distinguished from Sham cells after the Uniform Manifold Approximation and Projection (UMAP)-based dimensionality reduction, we used two-sample Wilcoxon rank test to measure the differences in the two UMAP coordinates between TBI and Sham cells. Specifically, ependymal cells had the highest statistical significance at UMAP-2 (Figure 5A, *p*-value < 0.05). The sub-clustering analysis of ependymal cells revealed two subclusters, namely C0 and C1 (Figure 5B). Notably, 94.3% of C0 ependymal cells are from TBI, while 63.38% and 36.62% of C1 ependymal cells are from TBI and Sham, respectively, suggesting that C0 might be TBI-specific ependymal cells (Figure 5C). The scRNA-seq-based differential gene expression analysis revealed that transcriptomes of ependymal cells in TBI greatly differed from those of Sham cells (Figure 5D). Gene set overrepresentation enrichment analysis (ORA) revealed that an enhanced activity of transition metal ion homeostasis and an attenuated activity of cilium movement were observed in the TBI ependymal cells (C0 cluster) had (Figure 5E). As ependymal cilium could maintain cerebrospinal fluid flow (Xiong et al., 2014), these results indicated that the attenuated activity of cilium movement after TBI might decrease cerebrospinal fluid flow.

### Comparative Analysis of the Transcriptional Changes Between Cortical and Hippocampal Traumatic Brain Injury Samples

**FIGURE 6:**
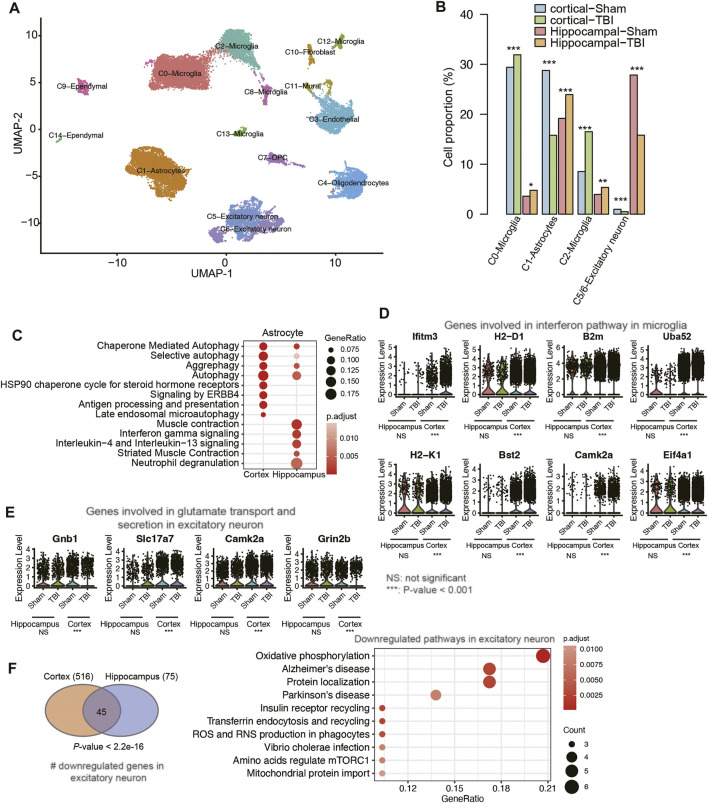
The differences of transcriptional changes between cortical and hippocampal TBI samples. **(A)** The UMAP analysis for the mouse cortical and hippocampal samples. Each point represents one cell, and the point colors represent the cell clusters. **(B)** The differential cell proportions between TBI and sham samples from cortex or hippocampus tissues. **(C)** The pathways enriched by the upregulated genes in the astrocyte of cortical and hippocampal TBI samples. **(D)** The expression of genes involved in interferon pathway in microglia. **(E)** The expression of genes involved in glutamate transport and secretion in excitatory neuron. **(F)** The pathways enriched by the downregulated genes in the excitatory neuron of both cortical and hippocampal TBI samples.

To investigate the concordance and discordance in transcriptome changes between cortex and hippocampus after TBI, we compared the scRNA-seq data of the hippocampal TBI samples with cortical samples [GEO accession: GSE160763 (Witcher et al., 2021), two vehicle TBI and two vehicle Sham samples]. Through the integration of those scRNA-seq samples and cell clustering, we also identified 10 cell types using the aforementioned marker genes (Figure 6A). The cell proportion analysis indicated that the proportions of microglia and excitatory neurons were increased in both hippocampal and cortical TBI samples, compared with the corresponding Sham samples; however, the proportion of astrocytes was decreased in cortical TBI samples, contrary to observations in hippocampal TBI samples (Figure 6B). In addition, we also speculated the proportion of activated astrocyte (*Gfap* ^+^), and found that *Gfap* ^+^ astrocytes were increased in the both cortical and hippocampal TBI samples (cortex, TBI vs. Sham: 3.92% vs. 2.55%; hippocampus, TBI vs. Sham: 16.46% vs. 11.99%), suggesting that astrocyte activation was observed in both cortex and hippocampus after TBI. The pathway enrichment analysis revealed that autophagy-related pathways were activated in the astrocytes of both hippocampal and cortical TBI samples, while the inflammatory pathways were more significantly enriched in the astrocytes of hippocampal samples (Figure 6C), suggesting that it was in the hippocampal astrocytes that these inflammatory pathways were activated by TBI rather than in the cortical astrocytes.

Furthermore, we also conducted such analyses on microglia and excitatory neurons. While a previous study reported that interferon pathway was activated in microglia after TBI (Witcher et al., 2021), the comparative analysis suggested that the expression levels of genes involved in interferon pathway were not significantly altered between TBI and Sham samples in hippocampus (Figure 6D). By contrast, inflammatory pathways such as IL18 signaling pathway, neutrophil degranulation, chemokine receptor bind chemokines, NOD-like receptor signaling pathway, and TYROBP causal network were highly enriched by the upregulated genes in the microglia of hippocampal TBI samples (Supplementary Figure S1). Similarly, the genes involved in glutamate transport and secretion were upregulated in the excitatory neurons of hippocampal TBI samples, but their expressions remained unchanged in the excitatory neurons of cortical TBI samples (Figure 6E). By contrast, the downregulated genes in excitatory neurons from hippocampal and cortical TBI samples showed high consistency (Figure 6F, Fisher’s exact test, *p*-value < 2.2e-16), and were enriched in neurological disorders, such as Alzheimer’s disease and Parkinson’s disease, and energy metabolisms, such as oxidative phosphorylation and mitochondrial protein import (Figure 6F, adjusted *p*-value < 0.05). These results indicated that though transcriptional changes in hippocampal and cortical TBI samples had their own unique characteristics, commonalities between them could be identified and addressed.

### The Potential Molecular Basis of Angiotensin Receptor Blocker Candesartan in the Treatment of Traumatic Brain Injury

**FIGURE 7:**
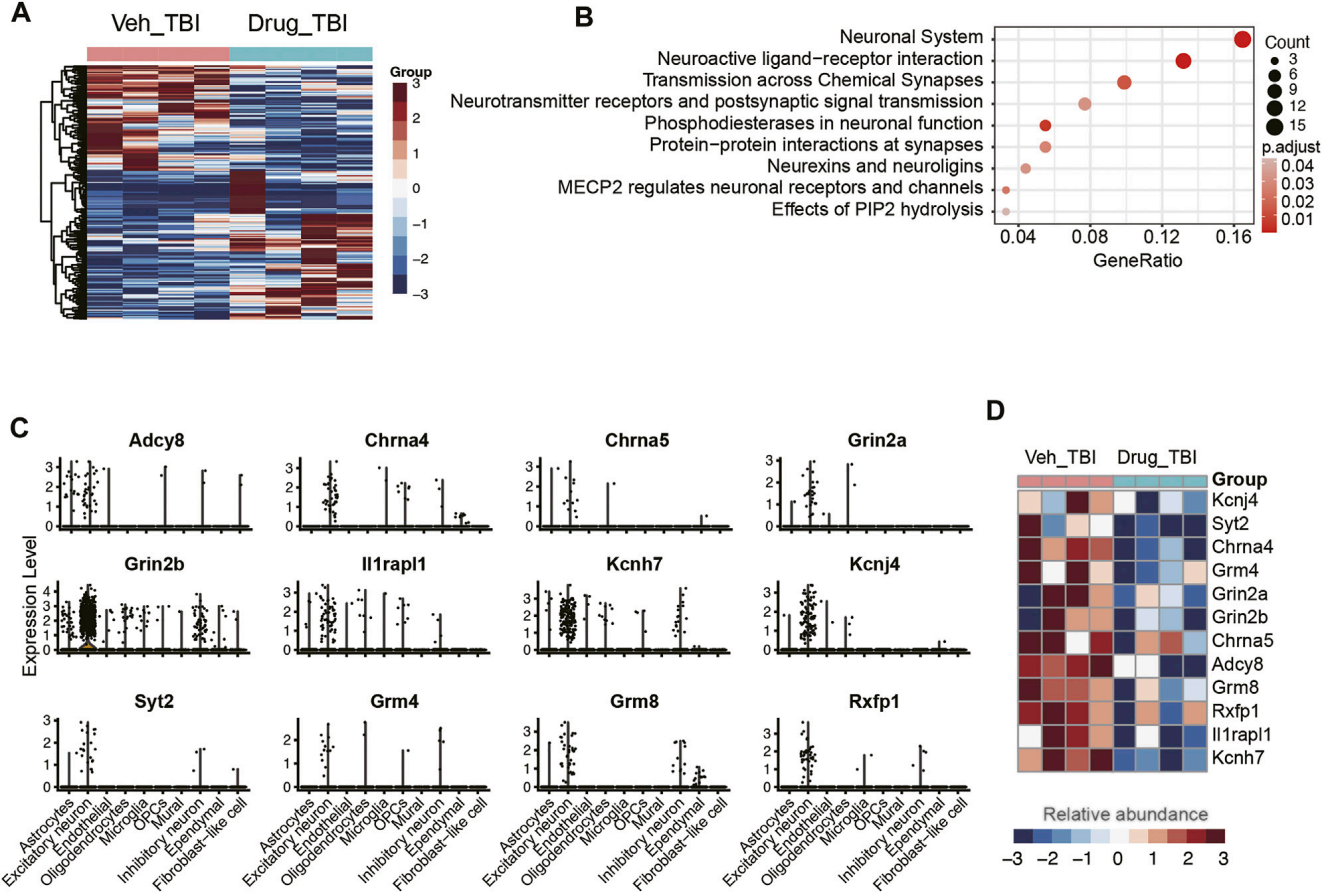
The association of excitatory neurons with angiotensin treatment. **(A)** The differentially expressed genes between the angiotensin-treated (Drug_TBI) and untreated (Veh_TBI) samples. **(B)** The pathways enriched by the downregulated genes of angiotensin-treated samples. The genes specifically expressed in excitatory neurons **(C)** were downregulated in angiotensin-treated samples **(D)**.

Previous studies have reported that candesartan, one of the angiotensin receptor blockers, could reduce lesion volume and improve motor and memory function after traumatic brain injury in mice (Villapol et al., 2012; Hajmohammadi et al., 2019; Attilio et al., 2021), however, the molecular mechanism is still poorly understood. To gain insight into the potential molecular basis of this drug, we conducted an integrative analysis of single-cell (GEO accession: GSE101901) and bulk RNA-seq data (Attilio et al., 2021) (GEO accession: GSE163415) to analyze the cell-type-specific changes in gene expression patterns. The differential expression analysis of the bulk RNA-seq data identified a total of 438 differentially expressed genes (DEGs) between hippocampus tissues of TBI with and without candesartan treatment (Figure 7A), including 243 upregulated and 195 downregulated genes in samples treated with candesartan. Subsequently, we found that the downregulated genes were primarily enriched in pathways like neuronal system and neuroactive ligand-receptor interaction (Figure 7B). Notably, the downregulated neuroactive ligands, receptors, or regulators involved in neuronal system, such as *Adcy8*, *Chrna4*, *Chrna5*, *Grin2a*, *Grin2b*, *Il1rapl1*, *Kcnh7*, *Kcnj4*, *Syt2*, *Grm4*, *Grm8*, and *Rxfp1*, were specifically expressed by excitatory neurons after candesartan treatment, suggesting that candesartan might relieve the symptoms of TBI by reducing the cellular excitotoxicity (Figures 7C,D). These results indicated that candesartan might promote recovery after traumatic brain injury *via* mediating the neuroactive ligand-receptor interactions and reducing cellular excitotoxicity.

## Discussion

Traumatic brain injury (TBI) is currently a substantial public health problem and one of the leading causes of morbidity and mortality worldwide (Dewan et al., 2018). However, the transcriptional changes after TBI at single-cell level have not been well characterized.

In this study, we identified 10 cell types, including astrocytes, endothelial cell, ependymal cell, excitatory neuron, fibroblast-like cell, inhibitory neuron, microglia, mural, OPCs, and oligodendrocyte utilizing scRNA-seq data. Among these cell types, oligodendrocyte accounted for the highest proportion of total cells, which was consistent with the observation that oligodendrocytes proved to be the most abundant glial cells in the mouse brain in a previous study (Valério-Gomes et al., 2018). The sub-clustering analysis identified three oligodendrocyte subpopulations (C0-C2), that were characterized by apoptotic, differentiated, and intermediate early states, respectively. The trajectory analysis also indicated that the oligodendrocytes might develop from C2 to C1, eventually to C0. In this study, we calculated the cell proportions for each sample and compared the cell proportions between TBI and Sham groups using *t* test, and our findings were inconsistent with the previous scRNA-seq study where both oligodendrocytes and OPCs were significantly decreased post-TBI (Arneson et al., 2018).

The differential cell proportion analysis revealed that astrocyte populations were significantly expanded in TBI samples. Moreover, the astrocytes were activated in TBI, as *Gfap*, a marker of astrocyte activation and a hallmark of multiple central nervous system (CNS) pathologies (Sofroniew, 2009), was significantly upregulated in TBI. Moreover, inflammatory response-related pathways, including signaling by interleukins, IL-18 signaling pathway, and interleukin-4 and interleukin-13 signaling pathways were significantly upregulated in the astrocytes in TBI samples. As microglia were well-recognized as major resident immune cells of the brain (Ginhoux et al., 2013), we also investigated their transcriptional changes after TBI. Consistently, inflammatory pathways such as IL18 signaling pathway, neutrophil degranulation, chemokine receptor bind chemokines, NOD-like receptor signaling pathway, and TYROBP causal network were highly enriched by those upregulated genes in the microglia in TBI samples (Supplementary Figure S1). These results suggested that both astrocytes and microglia were responsible for the inflammation in the acute phase of TBI.

As cellular excitotoxicity is a key mediator in the pathophysiology of TBI (Ng and Lee, 2019), we found that the upregulated genes in excitatory neurons of TBI were involved in hormone secretion, transport, and exocytosis. Considering that the release of glutamate was upregulated after TBI (Guerriero et al., 2015), we speculated that excitatory neurons might excessively transport and excrete glutamate in response to TBI. In contrast to a previous study that found CA1 neurons had higher expression levels of glutamate transporters and had the potential to differentiate or self-renew (Arneson et al., 2018), we found a neuron subpopulation as excitatory neurons, which presented an increase in the expression levels of genes involved in hormone transport and secretion and exocytosis. The sub-clustering analysis of ependymal cells revealed two subclusters C0 and C1. Notably, C0 was significantly enriched in TBI samples and characterized by enhanced activity of transition metal ion homeostasis and attenuated activity of cilium movement. As ependymal cilium could maintain cerebrospinal fluid flow (Xiong et al., 2014), we speculated that the attenuated activity of cilium movement after TBI might decrease cerebrospinal fluid flow.

To interpret the potential molecular basis of angiotensin receptor blocker, candesartan, for treating TBI, we conducted an integrative analysis of bulk and single-cell RNA-seq data to analyze cell type-specific gene expression changes. Specifically, we observed that downregulated genes were preferentially expressed by excitatory neurons in response to candesartan treatment, and were involved in pathways like neuronal system and neuroactive ligand-receptor interaction. These results indicated that candesartan might promote recovery after traumatic brain injury *via* mediating the neuroactive ligand-receptor interactions and reducing cellular excitotoxicity.

Compared with the previous single-cell studies (Arneson et al., 2018; Witcher et al., 2021), our study had three main distinctions. Firstly, we employed different statistical methods for cell proportion comparison between TBI and Sham groups. Specifically, we calculated the cell proportions for each sample (three Sham and three TBI replicates) and compared the cell proportions between TBI and Sham groups using *t* test, while the previous study (Arneson et al., 2018) merged the replicates into TBI or Sham groups and compared their cell proportions without considering the variance between samples when calculating the merged cell proportions. Secondly, we also observed concordance and discordance in the transcriptome changes of astrocyte, microglia and excitatory neurons between cortex and hippocampus after TBI. Specifically, astrocyte activation was more significant in hippocampus than cortex, and it was in the astrocyte of hippocampus that inflammatory pathways were activated by TBI rather than in that of cortex. The seemingly contradictory results that the proportion of astrocytes was increased in hippocampal cells but decreased in the cortex, as well as the differences of most abundant cell types between cortex and hippocampus, might be due to the differences in cell-type compositions between cortex and hippocampus. Particularly, the major cell types that were primarily increased in TBI cortex and hippocampus are microglia and astrocyte, respectively. Moreover, as we measured the relative proportions of different cells, it can be inferred that the dramatic increase of microglia proportion might result in a decreased proportion of astrocyte in TBI cortex. In addition, inflammatory pathways were activated in the microglia of both hippocampal and cortical TBI samples; however, interferon pathway was only activated in the microglia of cortical TBI samples. Glutamate transport and secretion were activated in the excitatory neurons of hippocampal TBI samples, but they remained unchanged in the excitatory neurons of cortical TBI samples. Thirdly, we also explored the molecular basis of angiotensin receptor blocker candesartan in the treatment of TBI, and found that candesartan might promote recovery after traumatic brain injury *via* mediating the neuroactive ligand-receptor interactions and reducing cellular excitotoxicity.

In conclusion, our study identified key cell types involved in and/or responding to TBI, which improved our understanding of the cellular and transcriptional changes after TBI, and of the molecular mechanisms that could serve as therapeutic targets.

## Data Availability

The original contributions presented in the study are included in the article/Supplementary Material, further inquiries can be directed to the corresponding author.
