# Supplementary figures and images for: Single-Cell RNA Sequencing Reveals Cellular and Transcriptional Changes Associated With Traumatic Brain Injury
Source: Front Genet. 2022 Jun 30;13:861428. doi: 10.3389/fgene.2022.861428 (PMC9282873; doi:10.3389/fgene.2022.861428)

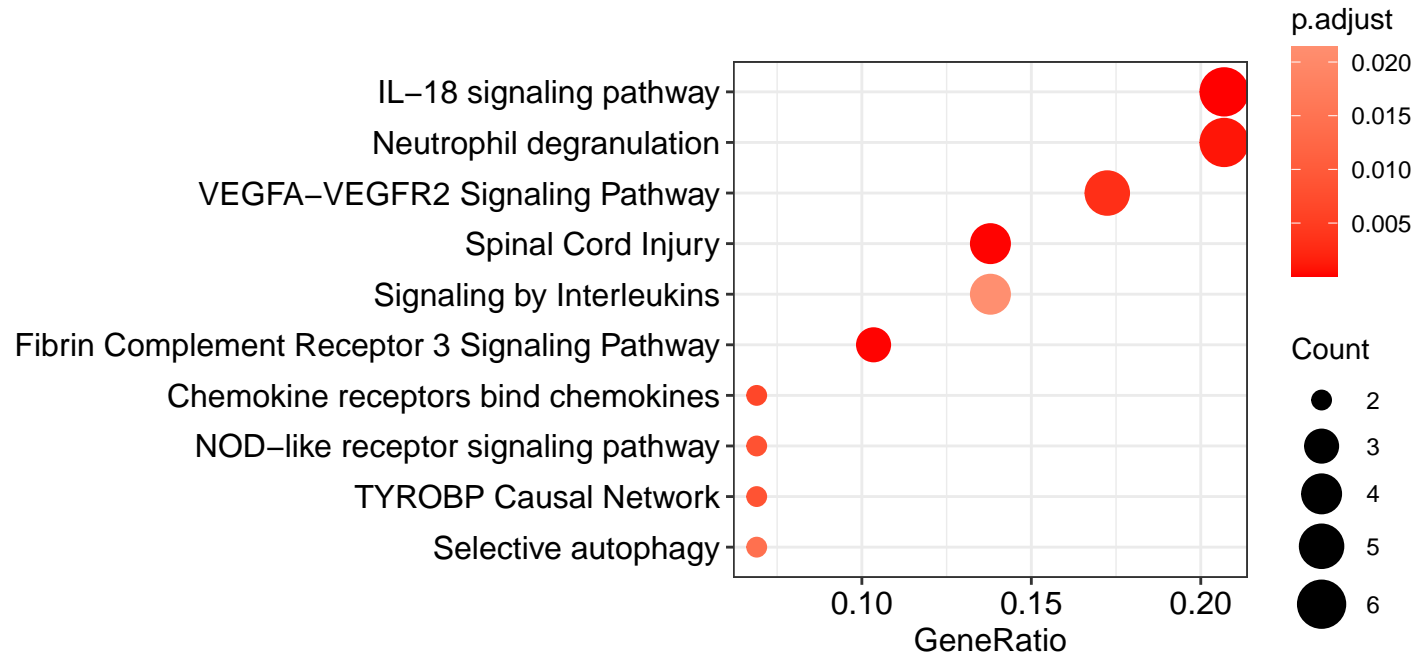

Supplement: Supplementary file 1 [file Image1.pdf]
